# EXPLORING THE OPTIMIZATION PATH OF LONG-TERM CARE INSURANCE SYSTEM IN RURAL AREAS OF CHINA

**DOI:** 10.1093/geroni/igae098.2795

**Published:** 2024-12-31

**Authors:** Hong Mi, Wanwan Si

**Affiliations:** Zhejiang University, Hangzhou, Zhejiang, China (People’s Republic); Zhejiang University, Hangzhou, Zhejiang, China (People’s Republic)

## Abstract

With the deepening of population aging and changes in family structure, the care needs of the disabled and mentally retarded older people have increased, and the problem of rural aging population has become more serious. In the face of the increasingly serious problem of rural aging, the improvement of the long-term care insurance system is the key to solving the problem of older people care in China. This paper focuses on the optimization path of the long-term care insurance system in rural areas of China, and researches from the aspects of the scope of coverage, assessment of incapacitation and payment of benefits, the management of the care model, and the content of the service. By analyzing the cost and expenditure situation of long-term care insurance in rural areas and the sustainability of its pilot program, combined with the data on the actual operation of long-term care insurance, this paper establishes a population-fund prediction model for the long-term care system, discusses the balance of inputs between medical care and daily life care, and provides targeted theoretical references and practical guidance for improving and perfecting the long-term care insurance system in rural areas of China.

